# Understanding the Workplace-Violence-Related Perceptions and Coping Strategies of Nurses in Emergency Rooms

**DOI:** 10.1097/jnr.0000000000000581

**Published:** 2023-11-28

**Authors:** Hsiu-Fen HSIEH, Yao-Mei CHEN, Shu-Lin CHEN, Hsiu-Hung WANG

**Affiliations:** 1 PhD, RN, Associate Professor and Adjunct Research Fellow, School of Nursing, College of Nursing, Kaohsiung Medical University, Taiwan; 2 PhD, RN, Assistant Professor, School of Nursing, College of Nursing, Kaohsiung Medical University, Taiwan; 3 MSN, RN, Head Nurse and Instructor, Department of Nursing, Kaohsiung United Municipal Hospital, Taiwan; 4 PhD, RN, FAAN, Professor, College of Nursing, Kaohsiung Medical University, Taiwan.

**Keywords:** workplace violence, emergency nurses, perceptions, coping strategies, qualitative research

## Abstract

**Background:**

Workplace violence (WPV) is a well-known and serious issue in most countries, and WPV against healthcare providers is of particular concern, especially among nurses working in emergency rooms (ERs).

**Purpose:**

We aimed to develop a deeper understanding of nurses' perceptions and coping strategies related to WPV that took place over a 1-year period from the perspective of nursing victims still working in ERs in southern Taiwan.

**Methods:**

This is a qualitative study with in-depth and semistructured interviews. Nineteen ER nurse victims were recruited from six hospitals in southern Taiwan from June 2015 to April 2016. All of the interview recordings were analyzed using content analysis.

**Results:**

The content analysis identified two themes of perceptions and two themes of coping strategies toward WPV. The two themes of perceptions were “adversity” and “dilemma,” with the former covering the three subthemes of “misunderstanding of health policy,” “unsafe environment,” and “nursing shortage” and the latter covering the two subthemes of “burnout” and “keeping or quitting the job.” The two themes of coping strategies were “adjustment” and “resilience,” with the former covering the three subthemes of “acceptance of the reality of WPV,” “self-regulation,” and “culture and belief” and the latter covering the two subthemes of “living with WPV” and “problem solving.”

**Conclusions/Implications for Practice:**

The findings revealed that ER nurse victims of WPV experienced a complicated journey after encountering WPV. Their coping strategies may be referenced by other ER nurses to better prevent and manage violent events in ERs. To prevent and manage violence in ERs, hospital managers should create a safe working environment through, for example, assigning sufficient security personnel and staff; provide relevant training to ER nurses in communications and other skills; and implement support systems to strengthen nurse resilience.

## Introduction

Workplace violence (WPV) is an important concern that greatly impacts the physical and mental health of its victims (World Health Organization [WHO], 2012). Hospitals are frequent targets of WPV. Serving on the healthcare front lines, nurses spend more time on patient care than other healthcare professionals, which makes them more likely to encounter WPV than other healthcare providers (Chapman et al., 2010). WPV committed by patients and their families in hospitals is a global problem with increasing organizational and social costs (Alkorashy & Al Moalad, 2016; Wei et al., 2016). WPV was defined by the Occupational Safety and Health Administration, Ministry of Labor, Taiwan, ROC (2016) as any act or threat of physical violence, harassment, intimidation, or other threatening disruptive behaviors occurring at a worksite. For emergency room (ER) nurses, the potential risk of being a victim of violence may induce a sense of despair and fear, which may negatively affect the quality of nursing care subsequently provided to their patients (International Council of Nurses, 2009; WHO, 2003).

The rising rate of WPV in healthcare systems has become a major problem for healthcare providers, mainly nurses (WHO, 2012) and ER nurses in particular (Vezyridis et al., 2015). The Emergency Nurses Association (2014) regards WPV as a serious occupational hazard for ER nurses. One study estimated that 74.1% of ER nurses experienced verbal abuse and 18.5% experienced both verbal and physical violence over a 1-year period (Alyaemni & Alhudaithi, 2016). In another, 88% of ER nurses self-reported as experiencing WPV during the previous 6-month period (Copeland & Henry, 2017). ER nurses in Taiwan face a relatively higher risk of encountering WPV than nurses in other specializations (Lee et al., 2010; F.-Y. Lin & Huang, 2016). Previous studies in Taiwan have shown the incidence of WPV in ER settings has increased in recent years, with rates of WPV ranging from 53% to 75% (Lee et al., 2010; F.-Y. Lin & Huang, 2016).

In Taiwan, although it is very convenient for people to obtain healthcare services under the National Health Insurance Scheme (National Health Insurance Administration, 1995), this system has created many problems for healthcare providers. For example, some recipients of care do not understand or accept that “the priority for emergency services is based on the critical condition of the patient.” Some patients with minor ailments are not satisfied because they are not treated expeditiously and wait for longer periods than expected. Their frustration may lead to their becoming violent and assaulting healthcare providers (Lee et al., 2010; Yang et al., 2015). Usually, patients go to ERs in response to serious or life-threatening situations. However, patients with symptoms of alcohol or drug abuse may exhibit violent behaviors, and they and their families may exhibit such behaviors after waiting for a longer period than expected for treatment (Vezyridis et al., 2015). Nurses are on the front lines of healthcare and thus spend more time on patient care than other healthcare professionals, making them more prone to encounter/experience WPV (Ferns, 2007).

The results of prior studies indicate patients' rights and violence may be related (Aivazi et al., 2017). The rights of patients in Taiwan have expanded significantly in recent years, and some patients expect to receive more service than they actually need. When patients' rights or their expectations cannot be immediately satisfied by ER medical staff, violence can take place. The imbalance in rules for medical treatment between healthcare providers and patients combined with ineffective communication may also be a major cause of WPV (Y. H. Lin & Liu, 2005; Magnavita, 2014). The safety and dignity of emergency nurses are not being adequately respected.

The consequences of WPV in its victims include low self-esteem, anxiety, and posttraumatic stress syndrome (Gong et al., 2014), poor morale and depression (Abraham et al., 2018; Edward et al., 2014), and symptoms characteristic of burnout (Chen et al., 2016). Notably, WPV increases emotional disturbance, turnover rates, and burnout in nurses and causes poor quality of sleep, all of which decrease job satisfaction (Lee et al., 2010; Taiwan Radical Nurses Union, 2014). It is necessary to take precautions and to implement an organizational structure against WPV in ER settings. The ability to recover from violent events, that is, resilience, varies among victims of WPV (DiCorcia & Tronick, 2011). Most people in Taiwan accept the concept of “holding good thoughts, doing good and accumulating merit” and are inspired and soothed by religious beliefs when facing adverse circumstances (Chang, 2009; Yee & Liu, 2004). These cultural traits help encourage ER nurses who experience WPV to continue working in the ER.

Most of the literature related to WPV has focused on quantitative studies related to types, prevalence, and mental and physical problems. Because of the limited qualitative studies on the coping strategies used by victims of WPV, in-depth interviews with victims who remained at their jobs were used to collect study data. Furthermore, a deeper understanding of the experiences, perceptions, and coping strategies of ER nurse victims of WPV is essential to prevent and manage WPV in the future.

The aim of this study was to elucidate the WPV-related perceptions and coping strategies of ER nurse victims of WPV that occurred during the past 1-year period.

## Methods

### Study Design

In this qualitative study, data were collected using in-depth interviews and relevant, probing questions (Minichiello et al., 1995) to obtain a comprehensive understanding of the perceptions and coping strategies of ER nurse victims of WPV.

### Settings and Participants

Purposive snowball sampling was used to recruit participants from six hospitals. Several head nurses in the Emergency Medical Network in southern Taiwan introduced qualified participants to the principal investigator, after which the participants introduced other potential participants. Inclusion criteria included nurses who (a) had worked for at least 3 months in the ER and (b) had experienced verbal or physical violence in the workplace during the past year. The exclusion criteria included nurses who (a) had experienced a stressful life event (e.g., death of a spouse/child, divorce) during the past year and (b) had a preexisting psychiatric disorder.

### Data Collection

Data were collected from June 2015 to April 2016. The principal investigator adjusted the direction and depth of the interviews based on each participant's situation and response and tried not to affect or interrupt their thoughts or emotional expressions during the interview. Each participant was interviewed for approximately 60–90 minutes. The principal investigator wrote an interview summary and showed it to each participant after the interview for mutual confirmation. Data were collected until saturation was achieved.

### Ethical Considerations

The interview dates, times, and locations were chosen for the convenience of the participants. Except for the informed consent forms, in which names and codes were delinked, all of the data, including field diaries, audio recordings, and text transcripts, were encoded to maintain participant confidentiality. All of the participants agreed to have their interviews audio recorded, and the text transcripts were carefully stored by the researchers. Before commencing this study, ethics committee approval (Institutional Review Board No. KMUHIRB-E-20150049) and institutional permissions were obtained. The participants were informed about the study purposes, and their written consent to participate was obtained before being interviewed.

### Data Analysis

Content analysis was used to interpret the transcribed text (Krippendorff, 2004). Following the guidelines for qualitative research interviews of DiCicco-Bloom and Crabtree (2006), we developed the interview guide for this study based on a literature review (Abraham et al., 2018; Yang et al., 2015). During the interviews, we asked the questions outlined in Table 1 regarding the participants' perceptions and coping strategies related to their WPV experiences.

**Table 1.** Interview Questions

No.	Question
1.	In what kind of situation did you experience violence?
2.	What type of violence did you suffer?
3.	What were your reactions to and perceptions regarding this violence?
4.	How did you cope with this violence?
5.	Have you ever asked for related help afterward?
6.	What kind of related assistance did you need most afterward?

All audio recordings were transcribed into verbatim transcripts, and then the text transcripts were examined carefully to ensure adequate depth and breadth. Thereafter, content with similar semantics were summarized using codes, which were subsequently grouped based on the similarity of features until themes were formed (Sandelowski & Barroso, 2003). The transcriptions were reviewed, and panel discussions were held three times until coding consensus was reached. On the basis of the review process and panel discussions, meaningful units were identified, and initial codes were extracted. As the analysis continued, initial themes and subthemes emerged as the similarities and differences in the codes were compared.

### Rigor and Trustworthiness

The principal investigator, the first author and the person responsible for collecting all of the study data, has over 20 years of psychiatric clinical nursing experience and has conducted various research projects in this field that have been published in the literature. The corresponding author has previously conducted research in the field of WPV and has published several qualitative research articles. The panel discussion team included a nursing educator and nursing manager with a PhD degree and qualitative research experience and a senior nurse with a master's degree and over 20 years of clinical experience in the ER. On the basis of the parallel perspective method, study rigor was established using the following criteria: credibility, dependability, confirmability, and transferability (Lincoln & Guba, 1985).

#### Credibility

To achieve credibility, further enquiries were made, and unclear viewpoints were clarified during the face-to-face interviews. To ensure all of the intended meanings of the participants were understood, field notes were taken during discussions with each participant. At regular monthly conferences, the contents of the interviews and code names were discussed. All of these contributed to improving study credibility.

#### Dependability

The researchers listened to the audio recordings at least three times to enhance correctness. All of the analyzed data were reviewed repeatedly and cross-checked by two of the researchers to achieve coding consensus. To ensure consistency, we conducted a peer check of the text transcripts with the audio recordings. Two weeks after data analysis, several paragraphs were randomly selected from the text transcripts and reexamined using content analysis.

#### Confirmability

An open attitude was adopted during the participant interviews to reduce bias and ensure mutual confirmation of semantic meanings to reach a comprehensive understanding of participants' perceptions of and coping strategies for their WPV experiences. In addition, a summary of their interview content was shown to each participant to obtain mutual confirmation.

#### Transferability

To increase sample diversity, the participants were selected purposively. In addition, detailed descriptions of the enquiry were made to achieve transferability.

## Results

Although male ER nurse victims of WPV were not excluded, no male nurses were enrolled as participants. Sixteen of the 19 female ER nurses who participated in this study were head nurses, nurse leaders, or senior nurses. Their mean work experience was 11.6 years, and their average age was 33.6 (25–47; *SD* = 7.0) years (Table 2). The procedure used to analyze the data is presented in Table 3.

**Table 2.** Demographic Characteristics of the Participants (*N* = 19)

Variable	*n*	%
Age (years; *M* and *SD*)	33.6	7.0
Level of education		
< College	5	26.3
≥ College	14	73.7
Marital status		
Single, never married	11	57.9
Married or living with partner	8	42.1
Religion beliefs		
No	4	21.1
Yes	15	78.9
Experience in nursing (years; *M* and *SD*)	11.6	6.8
Workplace		
Medical center (one head nurse and seven staffs)	8	42.1
Religion-based hospital (five head nurses and six staffs)	11	57.9

*Note.* The age range is 25–47 years; the nursing experience range is 2.5–25 years.

**Table 3.** Themes and Subthemes Identified by Content Analysis

Concept	Theme	Subtheme	Example of Participants' Narratives
**Perception**: Participants were fully aware of the violent events, as reflected in their subjective unpleasant feelings, emotions, and perspectives.	Adversity	Misunderstanding of health policy	*Some patients or their families believe that they have paid the fee for national health insurance, so they have the right to get any medical service….*
		Unsafe environment	*Inappropriate arrangement of the space in the ER led to interference between patients and nurses.*
		Nursing shortage	*All the other staff were too busy to help me when the patient grabbed her high heels and used them to hit me.*
	Dilemma	Burnout	*…I was resentful at the way I had been treated violently at the workplace with job burnout.*
		Keeping or quitting the job	*I was severely affected as a result of the violence and I wanted to leave this job at that time. But when I saw that my colleagues kept working hard….*
**Coping strategies:** As the participants mentally processed their experiences, they began adjusting their attitudes and thought about how to obtain related resources, improve communication skills, and enhance their ability to deal with workplace violence.	Adjustment	Acceptance of the reality of workplace violence	*I was very angry at the time of being assaulted, but I think that violent reactions might be part of the patient's underlying disease….*
		Self-regulation	*The violent event reminded me what I had learned and forgotten, such as more appropriate communications with ER patients and their families and the skills to deal with violent events.*
		Culture and belief	*My belief let me know that “good mind, good find.” I'll confront it in a positive manner when I faced to those violent patients.*
	Resilience	Living with workplace violence	*If we let patients and their families know detailed information about treatment procedures, there will be less misunderstanding between the staff and the patients, and their anxiety about uncertainty will be reduced, too. It might be helpful to prevent the occurrence of violence.*
		Problem solving	*When we get patients' complaints about long time waiting and the inefficiency of our nursing care, we should think how to solve it with shortened waiting time, provide better nursing care and improve ER environment for patients.*

**Figure 1.**
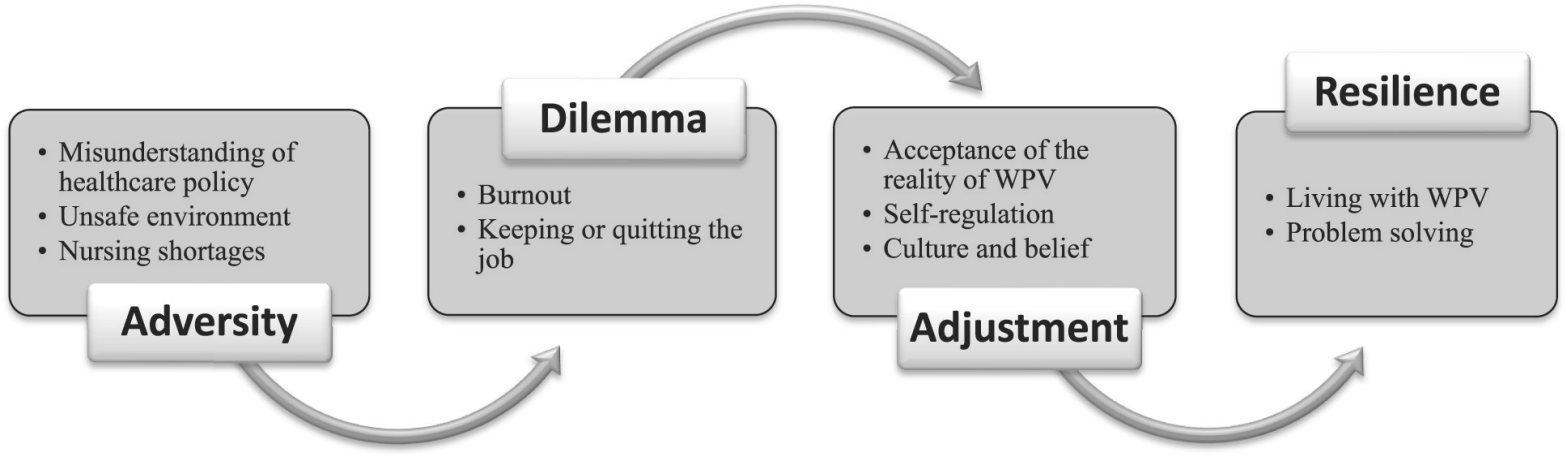
The Adjustment Process Undergone by the Participants *Note.* WPV = workplace violence.

The participants conveyed a variety of WPV-related perceptions and coping strategies. Two major perception themes, namely, “adversity” and “dilemma,” and coping strategy themes, “adjustment” and “resilience,” were respectively generated (Figure 1).

### Perceptions

The ER nurse victims of WPV were fully aware of the violent events, as reflected in their subjective unpleasant feelings, emotions, and perspectives. The participants' perceptions toward WPV included adversity (Theme 1) and dilemma (Theme 2).

#### Theme 1: adversity

Working in the ER was perceived by participants as a difficult and unpleasant job after being assaulted. This was regarded as “adversity” and included the subthemes “misunderstanding of health policy,” “unsafe environment,” and “nursing shortage.”

1. Misunderstanding of health policy

Approximately two thirds of the participants (12 of 19) reported that their unpleasant experiences with current healthcare policies and the ER environment were mainly associated with patients' insufficient knowledge of these policies and their inappropriate use of ER services. The lack of understanding among some patients and their families regarding the medical care hierarchy and the triage system was the source of conflicts between patients and healthcare providers.

Understanding of the ER triage system and medical care hierarchy should be promoted and strengthened publicly, as shown in the following participant quotes:

*Some patients or their families believe that they have paid the fee for national health insurance, so they have the right to get any medical service when they get sick or feel uncomfortable. Some patients without critical illnesses, such as those with fever or minor injuries, say that if we cannot relieve their discomfort instantly, they will make complaints to our managers about our poor quality of service and bad attitude*…. (Case H)

*Patients and their families don't know that the rule in the ER is that severe patients should be treated first, not that patients should be treated by order of arrival. They become irritable or even violent to ER nurses because of their dissatisfaction. No violence should occur in the hospital. We are helping them, not hurting them.* (Case F)

2. Unsafe environment

Approximately half of the participants (10 of 19) considered the ER environment to be imperfect and subject to risk of violence. They feel unsafe in the ER when interacting with patients and families with unstable emotions, as the following quotes illustrate:

*There was once a couple that came to the ER to manage the wife's wound which was caused by her husband. When we tried to separate them, the husband shouted at me and cursed at us with insulting words. Since he had enacted violence on his wife, he might assault me too. I was very scared at that time.* (Case D)

*Some unexpected conflicts were caused by disturbances between patients as a result of a poor partition design. Inappropriate arrangement of the space in the ER led to interference between patients and nurses, and such situations made the patients more irritable and violent*…. (Case M)

3. Nursing shortage

Approximately two thirds of the participants (12 of 19) expressed that WPV further aggravated the problem of nursing shortages, leading to a vicious cycle, as described below:

*The first time I thought about quitting my nursing job was when I was assaulted in the workplace. I finally understood why my ex-colleagues had left their jobs after suffering from WPV.* (Case E)

*I think that violence is one of the major causes leading to the nursing shortage.* (Case K)

*All the other staff were too busy to help me when the patient grabbed her high heels and used them to hit me. This made me so scared at that time.* (Case C).

#### Theme 2: dilemma

The participants expressed being extremely exhausted with burnout and experiencing decreased enthusiasm for working in the ER after encountering violence. In addition, they perceived a conflict between the ideal and the reality of their jobs and were uncertain about whether to continue helping patients or resigning to avoid further experiencing WPV.

1. Burnout

Approximately half of the participants (10 of 19) stated WPV had greatly impacted their physiological and psychological health. They felt disappointed and exhausted and thus wanted to leave their jobs as soon as possible, as illustrated in the following:

*I thought that I was strong and professional enough to be able to handle everything in the ER, and was confident about being violence-free. Unfortunately, I was wrong, and I eventually encountered violence in my workplace.* (Case C)

*Even though violent events were reported, it seemed nothing ever changed. I felt disappointed and exhausted. It was a tough time.* (Case I)

*I had never encountered such violence in the past, but it occurred in my workplace. At that time, I was resentful at the way I had been treated violently at the workplace, which led to job burnout.* (Case P)

*Zero tolerance for violence in the hospital should be the policy. I feel downhearted about this job.* (Case A)

2. Keeping or quitting the job

Nearly two thirds of the participants (12 of 19) expressed feeling perplexed and finding themselves at a crossroads after experiencing WPV. They wanted to leave their jobs to avoid experiencing such violence again. However, they also thought leaving their jobs would be equated with giving up and would only lead to further overloading their colleagues. The nurses described these sentiments as follows:

*I was severely affected as a result of the violence and I wanted to leave this job at that time. But when I saw that my colleagues kept working hard, I hesitated about the idea of leaving my job.* (Case N)

*After calming down, I had to face to the reality that I need this job and the salary for my life, even though I do not want to face violent patients or their families.* (Case O)

*On the one hand, I wanted to leave my job. On the other hand, leaving this job was equal to giving up my profession and would lead to overloading my colleagues.* (Case R)

### Coping Strategies

All of the participants in this study had remained working in the ER after their WPV experiences. As they mentally processed their experiences, they began adjusting their attitudes and thought about how to obtain related resources, improve communication skills, and enhance their ability to deal with WPV. The participants' coping strategies toward WPV included adjustment (Theme 3) and resilience (Theme 4).

#### Theme 3: adjustment

The participants considered violence to be inevitable in the ER, expressing their position through comments like “face it, accept it, and try to deal with it.”

1. Acceptance of the reality of workplace violence

All of the participants expressed being unhappy, sad, or angry during their WPV experience. More than three quarters (15 of 19) said that they had gradually changed from being angry to forgiving the WPV offenders, describing these sentiments as follows:

*I was very angry at the time of the assault, but I think that violent reactions may be part of the patient's underlying disease because hurting me was not his purpose for coming to the ER.* (Case I)

*Finally, I decided to keep this job and accepted the possibility of experiencing unexpected violence in the ER.* (Case D)

*Running away is not my style. I prefer to face and solve the problem by improving my communication skills.* (Case F)

2. Self-regulation

Approximately half of the participants (10 of 19) expressed feeling disappointment and exhaustion, and some stated they also had learned something positive from their WPV experiences:

*The violent event reminded me of things I had learned but forgotten such as the importance of using appropriate communications with ER patients and their families and learning skills to deal with violence events.* (Case Q)

*We hate WPV, but it seems to be part of the reeducation or continuing education materials in our professional field to make us stronger.* (Case S)

*There are always signs before violence erupts. I think that nurses' training needs to be strengthened. All frontline nurses must learn how to perceive in advance if there's anything wrong with a patient that could lead to violence. They need to warn themselves that perhaps violence is about to happen….* (Case A)

*As said by one of my seniors, working in the emergency room is also part of the training process. Sometimes you think you did well, but still got complaints. You feel upset, but you have to adjust your emotions.* (Case H)

3. Culture and belief

More than half of the participants (11 of 19) expressed being either Buddhist or Taoist. They perceived their religious faiths as helping them enhance specific thoughts that made them feel better after experiencing violence:

*My belief let me know that “good mind, good find.” I'll confront it in a positive manner when I face violent patients.* (Case C)

*As the old saying goes: “if one owed another in a previous life, sooner or later, he or she will have to pay back.” I may owe them a debt in a previous life, and I was paying them back at that time.* (Case I)

*I always keep it in my mind to “be a good person and do good deeds.” I regard it as “accumulating merit” by suffering from violence without asking for their responsibility.* (Case K)

#### Theme 4: resilience

After their WPV experience, the participants progressed from “adversity” to “dilemma” and, ultimately, “adjustment,” gaining the capacity to withstand, regulate, maintain equilibrium, and deal with violent events. This capacity is regarded as resilience.

1. Living with workplace violence

Because they had accepted the inevitability of WPV, the participants had decided to keep their job.

Approximately two thirds of the participants (12 of 19) had developed more-professional communication skills to help prevent future WPV incidents and to think positively about dealing with violence if it occurred again, as described below:

*Once, when I was caring for a patient, he suddenly hit me without any sign of violence (crying)…. I was scared and felt upset at that time. Then, I started to think that this nursing job might not suit me. Almost at the same time, some of my colleagues consoled me and gave me support. Then, I knew how to overcome the psychological obstacles, and I decided to keep doing my nursing job.* (Case M)

*Sometimes, we are anxious to treat patients in the ER, which may lead to patients receiving inadequate information and subsequent poor communications between patients and us. Now, I think if we had good communication skills, violent events can be avoided.* (Case N)

*If we let patients and their families know detailed information about treatment procedures, there will be less misunderstanding between staff and patients, and their anxiety over uncertainties will be reduced too. It could be helpful to prevent the occurrence of violence.* (Case J)

2. Problem solving

Most of the participants had received support and guidance from their colleagues, which strengthened their resilience and helped them recover after encountering WPV, as illustrated in the following:

*As said by one of my senior colleagues: working in the ER is part of the training process. Sometimes you think you did well, but you still get complaints from the patients. When you feel upset, your colleagues will give you support, and then you can go ahead with your job.* (Case H)

*When we get patients' complaints about long wait times and the inefficiency of our nursing care, we should think how to solve it through shorter waiting times, better nursing care, and improved ER environments for patients*. *Long waiting times with intractable pain may be a factor in violence. We should think how to shorten their waiting time.* (Case O)

## Discussion

The characteristics of the perceptions and coping strategies toward WPV experienced by ER nurses in Taiwan were explored in this study, with four themes and 10 subthemes extracted. From the perspective of the participants, WPV leads to heightened job adversity. ER nurses are the first responders for patients, and they face a particularly high risk of encountering violence committed by patients or their families (Yang et al., 2015). In addition, conflicts and disputes are more likely to occur in the ER than in other hospital departments because of the high number of unanticipated circumstances. ER patient treatment priority is based on the severity and urgency of each patient's condition. However, all ER patients believe they should be treated as quickly as possible (Hammad et al., 2018). There is lack of awareness among patients and their families about the hierarchy of medical care and the current triage system. In Taiwan, although obtaining healthcare services under the National Health Insurance system is convenient for patients (National Health Insurance Administration, 1995), it creates many problems for healthcare providers. For example, some people with minor or nonurgent illness go to the ER for quick treatment in violation of medical care hierarchy rules, leading to further overloading ER services. Another example is that some people do not understand or accept the rules of the current triage system, which prioritizes care based on the actual state of illness or severity of injury rather than on time of arrival or patients' self-perceptions regarding their condition. Some patients with minor ailments who do not have priority for emergency triage-based services are thus not satisfied with their quality of care because of waiting a long time and may become violent and assault healthcare providers (Lee et al., 2010; Yang et al., 2015).

The participants expressed feeling unsafe in the ER while interacting with patients or families with unstable emotions. They expressed that the designs of partitions and equipment in the ER were inappropriate or insufficient and exacerbated the negative emotions of the patients and their families. This result was similar to other studies (Isaak et al., 2017), supporting that a safer environment is needed for ER staffs. For example, adequately separated partitions can reduce interactions among patients, and providing ER nurses with personal alarms can help reduce WPV incidents (Gerberich et al., 2005).

Manpower shortages, a common phenomenon in nursing in Taiwan, is a direct cause of work overload, which often delays the provision of care to patients, increasing irritability and even violent reactions in some patients. WPV further aggravates nursing shortages, leading to a vicious cycle. Lower nurse–patient ratios significantly and positively impact quality of healthcare, reducing the risks of patient death and failure to recover (Aiken et al., 2014). To prevent violence and improve quality of care in hospitals, the nursing shortage problem should be solved by maintaining an adequate nurse–patient ratio. It has also been found that nurses who work in hospitals with better work environments have significantly higher job satisfaction and lower intention to leave and levels of burnout (Nantsupawat et al., 2017). The nurse–patient ratio in Taiwan ranges from 1:9 to 1:15, which is much higher than those in other advanced countries (Ministry of Health and Welfare, Taiwan, ROC, 2018). It has been suggested that the nurse-to-patient ratio should be decreased to 1:6 or lower.

In this study, the participants reported facing a dilemma after experiencing WPV. Similar findings were reported in another study in which women perceived their exposure to violence as being a dilemma in their lives (Schneider et al., 2017). Most of the participants in this study reported experiencing burnout, expressed through unhappiness, trouble sleeping, frustration, and feelings of hopelessness, after being assaulted. This finding corresponds with a previous study (Hamdan & Hamra, 2017) that found burnout to be positively associated with exposure to WPV. Burnout made these nurses want to leave their jobs, which was consistent with the results of other studies (Jones, 2017; Nantsupawat et al., 2017).

The participants reported feeling greater ambivalence toward their jobs after their WPV experience. Although many wanted to leave their jobs afterward, they ultimately perceived that quitting would equate to giving up on their profession and increasing the workload of other colleagues. A similar result was obtained in another study in which WPV was positively correlated with turnover intention and job burnout and negatively associated with job satisfaction (Duan et al., 2019). Upon retrospection, the participants had decided to keep their jobs in the ER and accepted the possibility of future WPV incidents committed by patients and their families, recognizing the ER as full of challenges and unusual/unanticipated situations that require their adjusting their own expectations and attitudes.

People in Taiwan generally seek help from their religious beliefs when facing adverse events/environments (Tsai, 2014). Most of the participants in this study expressed being influenced and comforted by their religious beliefs and allowing a “peaceful heart” to replace the negative emotions in their mind after their WPV experiences. Religious beliefs are an aspect of spirituality that provides people with spiritual support and consolation during and after adverse experiences such as WPV.

In this study, the participants expressed wanting to obtain relevant training to deal with violent events in their place of work. In another study, nurses who had been victims of WPV also expressed willingness to receive training to handle violent situations more appropriately (Zhou et al., 2017). Without effective communication between patients and healthcare providers, violent conflicts are likely to occur when patients have different understandings of or insufficient information about treatments or procedures (Yang et al., 2015). Consistent with other studies, most of the participants in this study considered improving their communication skills to be a good strategy for coping with WPV (Freeland et al., 2018; Yang et al., 2015).

Resilience plays an important role in improving the ability of ER nurses to deal with WPV. Resilience may be enhanced through peer support, helping reduce the risk of developing psychological problems after a WPV incident (Hsieh et al., 2016; Firmin et al., 2019). Factors such as peer support that enhance resilience should be bolstered in nurses to further enhance resilience.

### Limitations

This study was affected by several limitations. Although not excluded, no male nurses participated in this study. In addition, not all ER nurse victims of WPV continue working in the ER. The inclusion criteria did not permit us to explore the perceptions and coping strategies of those who had left their job after experiencing WPV.

### Conclusions/Implications for Practice

The findings of this study indicate ER nurses in Taiwan face a variety of professional challenges, including WPV. Resilience is critical to help ER nurse victims of WPV progress through their psychological journey from adversity to recovery. To prevent violence, hospital managers should provide a safe environment for nurses through measures such as assigning sufficient security personnel, conducting video surveillance, and hiring sufficient numbers of staff. In addition, skills training should be provided to ER nurses to help them obtain critical support resources, improve their communication skills, and enhance their ability to deal with WPV. Support systems should be sustained for ER nurses to enhance their resilience, which is an essential factor in preventing WPV-induced psychological sequelae. As support systems were identified by the participants as effective in promoting positive adjustment and greater resilience to WPV, the study results may be referenced by policymakers seeking to improve the provision of support systems to ER nurses.
